# Antimicrobial, Antibiofilm, and Antioxidant Potentials of Four Halophytic Plants, *Euphorbia chamaesyce*, *Bassia arabica*, *Fagonia mollis*, and *Haloxylon salicornicum*, Growing in Qassim Region of Saudi Arabia: Phytochemical Profile and In Vitro and In Silico Bioactivity Investigations

**DOI:** 10.3390/antibiotics12030501

**Published:** 2023-03-02

**Authors:** Osamah Al Rugaie, Hamdoon A. Mohammed, Salman Alsamani, Sabri Messaoudi, Lotfi M. Aroua, Riaz A. Khan, Suliman A. Almahmoud, Abdulrahman D. Altaleb, Mansour Alsharidah, Musaad Aldubaib, Khalid A. Al-Regaiey, Kamal A. Qureshi

**Affiliations:** 1 Department of Basic Medical Sciences, College of Medicine and Medical Sciences, Qassim University, Unaizah 51911, Saudi Arabia; 2 Department of Medicinal Chemistry and Pharmacognosy, College of Pharmacy, Qassim University, Buraydah 51452, Saudi Arabia; 3 Department of Pharmacognosy and Medicinal Plants, Faculty of Pharmacy, Al-Azhar University, Cairo 11371, Egypt; 4 Department of Chemistry, College of Science, Qassim University, Buraydah 51452, Saudi Arabia; 5 Faculty of Sciences of Bizerte, Carthage University, Bizerte 7021, Tunisia; 6 Laboratory of Structural Organic Chemistry: Synthesis and Physicochemical Studies (LR99ES14), Department of Chemistry, Faculty of Sciences of Tunis, University of Tunis El Manar, Tunis 2092, Tunisia; 7 Department of Physiology, College of Medicine, Qassim University, Buraydah 51452, Saudi Arabia; 8 Department of Veterinary Medicine, College of Agriculture and Veterinary Medicine, Qassim University, Buraydah 51452, Saudi Arabia; 9 Department of Physiology, College of Medicine, King Saud University, Riyadh 11451, Saudi Arabia; 10 Department of Pharmaceutics, Unaizah College of Pharmacy, Qassim University, Unaizah 1911, Saudi Arabia

**Keywords:** antimicrobial, antibiofilm, antioxidant, bioactivity, halophytes, phytochemicals, plant extract, *Euphorbia chamaesyce*, *Bassia arabica*, *Fagonia mollis*, *Haloxylon salicornicum*, in silico molecular docking, *S. aureus tyrosyl*-tRNA synthetase

## Abstract

The current study aimed to investigate the phytochemical contents and antioxidant, antimicrobial, and antibiofilm activities of four halophytic plants, namely, *Euphorbia chamaesyce*, *Bassia arabica*, *Fagonia mollis*, and *Haloxylon salicornicum*, native to central Saudi Arabia. The alcoholic extract of *E. chamaesyce* was found to be the most potent in various bioactivities-based evaluations and rich in polyphenols and flavonoid secondary metabolites, with 68.0 mg/g and 39.23 mg/g gallic acid and quercetin equivalents, respectively. Among all plants’ extracts, the alcoholic extract of *E. chamaesyce* had the highest DPPH scavenging and metal chelating antioxidant activities at 74.15 Trolox equivalents and 16.28 EDTA equivalents, respectively. The highest antimicrobial activity of *E. chamaesyce* extract was found to be against *Shigella flexneri*, with a mean zone of inhibition diameter of 18.1 ± 0.2 mm, whereas the minimum inhibitory concentration, minimum biocidal concentration, minimum biofilm inhibitory concentration, and minimum biofilm eradication concentration values were 12.5, 25, 25, and 50 mg/mL, respectively. The LC-ESI-MS/MS analysis of the *E. chamaesyce* extract showed the presence of six flavonoids and ten phenolic constituents. The in silico binding of the *E. chamaesyce* extract’s constituents to *Staphylococcus aureus* tyrosyl-tRNA synthetase enzyme displayed −6.2 to −10.1 kcal/mol binding energy values, suggesting that these constituents can contribute to the antimicrobial properties of the plant extract, making it an essential medicinal ingredient. In conclusion, these results warrant further investigation to standardize the antimicrobial profiles of these plant extracts.

## 1. Introduction

Desertification and the spread of saline regions are global environmental challenges that have had a significant impact on the health and well-being of our planet and its inhabitants [1,2]. The increase in the desertification process and soil salinity allows a class of plants to uniquely survive, grow, and reproduce; this class of plants is called halophytes [3,4]. Halophytes are, therefore, widely distributed in all continents except Antarctica [5] and are primarily used by rural people and Bedouins as a part of their food, medicine, and livestock feed [6,7]. In Saudi Arabia, halophytes are widely distributed in the coastal and central areas of the kingdom [8,9]. Halophytic plants possess remarkable characteristics, including the ability to withstand high salt concentrations, strong winds, and high temperatures. These plants produce a diverse range of secondary metabolites, such as polyphenols and flavonoids, that help them survive in harsh environments. These secondary metabolites have been shown to have potential therapeutic properties, making halophytes an important source of medicinal compounds [5,6,7,10,11].

Several studies have reported halophytes’ chemistry and biological activities. It has been conclusively established that these plants are high-potential sources of various phenolic and flavonoid compounds and have markedly exhibited antioxidant, anticancer, and antimicrobial activities [10,11,12,13,14,15]. The phenolics and flavonoids are considered chemical constituents exhibiting antimicrobial and antibiofilm properties, which make them attractive candidates for new drug discovery for treating infections [16,17]. However, research on antimicrobial therapy, including flavonoids and polyphenolic substrates, is ongoing. In contrast, the antioxidant properties of these halophytes have made them strong and interesting agents for preventing oxidative damage in the cells and other body tissues. To this effect, various studies have been carried out on the antioxidant, antibacterial, and antibiofilm activities of different halophytic plants and their phenolics and flavonoid phytochemicals extracted from halophytic plant sources [10,11,12,13,14,15].

This attention to the antimicrobial agent’s discovery is embedded in global concern for the high mortality rate caused by various infections [18,19], wherein antibiotic resistance is a significant barrier to effective microbial control globally [20,21]. The production of biofilms by microorganisms is important for survival in harsh environments and to evade eradication. Biofilms are produced by the communities of microorganisms that are attached to surfaces in contact and produce a matrix of extracellular polymeric substances (EPS) that encase the microorganisms. This matrix, EPS, provides a protective barrier for shielding the microorganisms from environmental stresses, including resistance or inhibition by antibiotics and other antimicrobial agents [22]. In this context, a worldwide search for effective antimicrobial and antibiofilm agents from natural and synthetic sources is strongly encouraged, which has attracted the attention of several researcher classes worldwide, including natural products chemists, microbiologists, and drug discoverers. The phytochemical diversity of plant extracts, in many ways, can induce various antimicrobial mechanisms in living entities and may result in synergistic antimicrobial effects owing to the participation of many phytochemicals present in the plant extracts [23,24]. It has been established that phenolic acids and flavonoids also possess antimicrobial properties, in addition to other plants’ constituents [25,26]. In addition, the presence of well-known antioxidant compounds in plant extracts can also add significant value to the use of these plants for microbial controls and for treating infections.

The current study was designed in this context to investigate the phytochemical constituents and antimicrobial, antibiofilm, and antioxidant properties of four indigenous halophytes found in central Saudi Arabia. The plants included *Euphorbia chamaesyce*, *Bassia arabica*, *Fagonia mollis*, and *Haloxylan salicornicum*. Traditionally, these plants are reputed in folklore medicine and are used to ameliorate the conditions of various disorders, including skin diseases, digestive malfunctions, and respiratory problems [27,28,29,30,31,32,33,34,35,36].

*E. chamaesyce* is a small herb belonging to the family Euphorbiaceae, commonly known as “spurge”, and is found in tropical and subtropical climatic regions, as well as in the central dry areas of Saudi Arabia. It has been used as part of traditional medicine by herbalists and nomadic Bedouin communities to treat infections, wound healing, and a variety of other disorders, such as skin diseases, respiratory problems, and inflammation [27,30]. *B. arabica* is a small shrub belonging to the family Chenopodiaceae, commonly known as “jubain”, in the central Saudi Arabian region of Qassim. The plant is also prevalent in the arid and semi-arid parts of the world. It is also part of local traditional medicine for treating diabetes, inflammation, wounds, and symptomatic liver malfunctions, including jaundice [32]. The *F. mollis* is a small shrub belonging to the family Zygophyllaceae, commonly known as “al-araq” in the Qassim region. The plant is also widely distributed in other dry and marshy areas of the kingdom. The plant is part of arid and semi-arid regions of the world. The plant has been traditionally used in folk medicine to treat various ailments, such as skin diseases, respiratory problems, and diabetes, by the populace in the local Qassim and other adjoining areas [33,34]. *H. salicornicum*, a small shrub, belongs to the family Amaranthaceae and is commonly known as “ghaf” in Saudi Arabian regions of the plant occurrence areas. The plant is commonly present in arid and semi-arid regions. The medicinally valued shrub is part of traditional medicinal chestnut and is used to treat different symptomatic diseases, such as troubled skin conditions, inflammation of joints, and digestive disorders [35,36].

Among the major Saudi Arabian halophytes, *E. chamaesyce*, *B. arabica*, *F. mollis*, and *H. salicornicum* are part of promising traditional medicinal plants species with therapeutic applications used over a long time by the desert-based nomadic Bedouin tribes and city dwellers of the country. The current study sheds light on these plants’ phytochemical, antimicrobial, antibiofilm, and antioxidant properties. It provides a basis for future investigations into the potential use of these plants in possibly developing new drug templates and drug candidates.

## 2. Results and Discussion

### 2.1. Total Phenolic Content (TPC), Total Flavonoid Content (TFC), and Total Antioxidant Activity (TAA) of the Plant extracts

Table 1 shows the results of the quantitative measurements of TPC and TFC for the aqueous ethanolic extracts of these four plants. Total phenolics and flavonoids were expressed as gallic acid equivalent (GAE) and quercetin equivalent (QE), and milligrams per gram of the dried extract. The results showed considerable quantities of phenolics and flavonoids in all four plant extracts. However, remarkably higher contents of these constituents were found in *E. chamaesyce*, i.e., 68.00 GAE and 39.23 QE, compared to other plants (Table 1). The lowest values for the phenolics and flavonoids were detected in the extract of *B. arabica*, which was analyzed at 45.32 GAE and 17.90 QE, respectively. In addition, the amount of phenolics and flavonoids in *H. salicornicum* and *F. mollis* were measured at 49.19 and 47.30 GAE and 19.33 and 24.79 QE, respectively. These results of the phenolics and flavonoids suggested that these classes of secondary metabolites are higher in concentration in the extract of *E. chamaesyce* as compared to the other three plants. The total phenolic content of *E. chamaesyce* aqueous extract has been previously reported as 26.6 GAE/g dry extract [37], which was significantly lower than the phenolic content in the ethanolic extract recorded in the current study (68.0 GAE of the dry extract). The higher phenolic content in *E. chamaesyce* may be primarily attributable to the effects of the harsh environmental conditions where the plant grows (central Saudi Arabia) than in the previous study’s location (Iran), which is comparatively better than the current conditions of the present study. However, a literature review suggested that *E. chamaesyce* possessed higher levels of phenolics and flavonoids, which is consistent with the present findings [38,39].

Table 1 also demonstrates the TAA (total antioxidant activity) of the ethanolic extracts of all the plants. The results indicated the higher antioxidant activity of *E. chamaesyce* compared to all other plants, consistent with the results obtained for the total contents of the phenolics and flavonoids in these plants. The extract of *E. chamaesyce* displayed free radical scavenging activity that was at least three times (3×) as high as that of other plant extracts, as evaluated at a value of 74.15 TE, as compared to the 18.46, 23.34, and 25.17 TE for the *B. arabica, F. mollis*, and *H. salicornicum* plant extracts, respectively. Additionally, the results of the ferric-reducing antioxidant power (FRAP) also indicated that the *E. chamaesyce* extract exhibited at least double the activity of other plant extracts in terms of its reducing power (Table 1). On the other hand, the metal chelating activity (MCA), which is mainly dependent on the structural characteristics of the phenolics and flavonoids rather than their quantities, was measured at very close values of 16.28, 16.58, 16.34, and 15.80 EE in *E. chamaesyce*, *B. arabica*, *F. mollis*, and *H. salicornicum*, respectively, indicating the structural commonality of these constituents. The overall antioxidant results revealed the potential antioxidant activity of all four plant extracts. The results unequivocally established the antioxidant activity superiority of the *E. chamaesyce* extract over all other plant extracts, which is predictable owing to the plant’s much higher levels of phenolics and flavonoids. The results are also consistent with the reported antioxidant activity of *E. chamaesyce* growing in different locations in the world [37,40].

### 2.2. Antimicrobial Profiles of F. mollis, B. arabica, H. salicornicum, and E. chamaesyce Plant Extracts

#### 2.2.1. Preliminary Antimicrobial Activity

Preliminary antimicrobial activity results showed that the ethanolic extracts of *E. chamaesyce*, *B. arabica*, and *H. salicornicum* had substantial antimicrobial activity; however, the *F. mollis* plant extract did not exhibit any antimicrobial strength. At the same time, no plant extracts showed antifungal activity against the tested microorganisms at the given dose of 5 mg of the plant extract. Furthermore, these results also showed that the *E. chamaesyce* plant extract had the strongest, *H. salicornicum* moderate, and *B. arabica* the lowest ranges of antimicrobial activity against the tested organisms at the given dose of 5 mg plant extract/disc (Table 2). As observed, the *E. chamaesyce* and *H. salicornicum* ethanolic extracts were highly effective against *S. flexneri* ATCC 12022, with mean ZIDs of 18.1 ± 0.2 mm and 13.3 ± 0.3 mm, respectively. In contrast, the lowest antimicrobial activity was observed against methicillin-resistant *Staphylococcus aureus * (MRSA), with mean ZIDs of 8.1 ± 0.2 mm and 6.9 ± 0.2 mm, respectively (Table 2). The *B. arabica* extract showed the highest antimicrobial activity against *Bacillus cereus* (*B. cereus*) ATCC 10876, with a mean ZID of 9.9 ± 0.2 mm. The lowest activity was observed against *Staphylococcus aureus* (*S. aureus*) ATCC 29213, with a mean ZID of 8.0 ± 0.2 mm. The negative control dimethyl sulphoxide (DMSO) exhibited no antimicrobial activity against all the tested organisms at a 20 µL/disc concentration (Table 2).

#### 2.2.2. MIC (Minimum Inhibitory Concentration), MBC (Minimum Biocidal Concentration), MBIC (Minimum Biofilm Inhibitory Concentration), and MBEC (Minimum Biofilm Eradication Concentration)

The four plants’ extracts showed varying levels of antimicrobial activity, as indicated by their MIC, MBC, MBIC, and MBEC values, as summarized in Table 3, Table 4 and Table 5. The *E. chamaesyce* extract had MIC values of 12.5–25.0 mg/mL, MBC values of 25.0–50.0 mg/mL, MBIC values of 25.0–50.0 mg/mL, and MBEC values of 50.0–100.0 mg/mL. The *H. salicornicum* extract had MIC values of 0.78–12.5 mg/mL, MBC values of 1.56–25.0 mg/mL, MBIC values of 1.56–25.0 mg/mL, and MBEC values of 3.13–50.0 mg/mL. The *B. arabica* extract had MIC values of 12.5–25.0 mg/mL, MBC values of 25.0–50.0 mg/mL, MBIC values of 25.0–50.0 mg/mL, and MBEC values of 50.0–100.0 mg/mL.

Several previous reports showed that the alcoholic extracts of various species of *Euphorbia* plants exhibited substantial antimicrobial potential against various pathogens [27,41]. Kirbag et al. reported that the methanolic extract of various species of *Euphorbia* plants exhibited substantial antimicrobial activity against various tested organisms. These results corroborated our findings that the *E. chamaesyce* extract had substantial antimicrobial potential against various human pathogens, including *S. aureus*, MRSA, *S. saptophyticus*, *B. cereus*, and *S. flexneri*. Reezal et al. reported that the aqueous and alcoholic extracts of *E. hirta* were effective in inhibiting the growth of all tested microbes, i.e., *E. coli*, *Salmonella enteritidis* (*S. enteritidis*), *S. aureus*, and *Bacillus subtilis* (*B. subtilis*). Concentration-dependent growth inhibition of *C. albicans* was also observed when leaf and bark aqueous extracts were used. The microorganism *C. tropicalis* was also sensitive to the aqueous leaf extract [27]. These reports corroborated our results that the alcoholic extract of *Euphorbia* exhibits substantial antimicrobial activity against currently tested organisms as present in our study. In addition, there is also the contradictory finding of antifungal activity in *E. hirta*, which was not observed from the *E. chamaesyce* ethanolic extract. This suggested that the difference in plant species, growing location, and environmental conditions can also play a part in exhibiting bioactivities.

#### 2.2.3. Statistical Analysis

The results of one-way ANOVA (analysis of variance) revealed that there was a statistically significant difference (*p* < 0.05) in the mean antimicrobial values among the groups of tested microbial strains, i.e., *E. chamaesyce*, F (11, 24) = 2506.561, *p* = 0.000; *H. salicornicum*, F (11, 24) = 756.165, *p* = 0.000; *B. arabica*, F (11, 24) = 667.193, *p* = 0.000 (Table S1 from Supplementary Materials).

### 2.3. LC-MS Analysis of E. chamaesyce Extract

**Figure 1:**
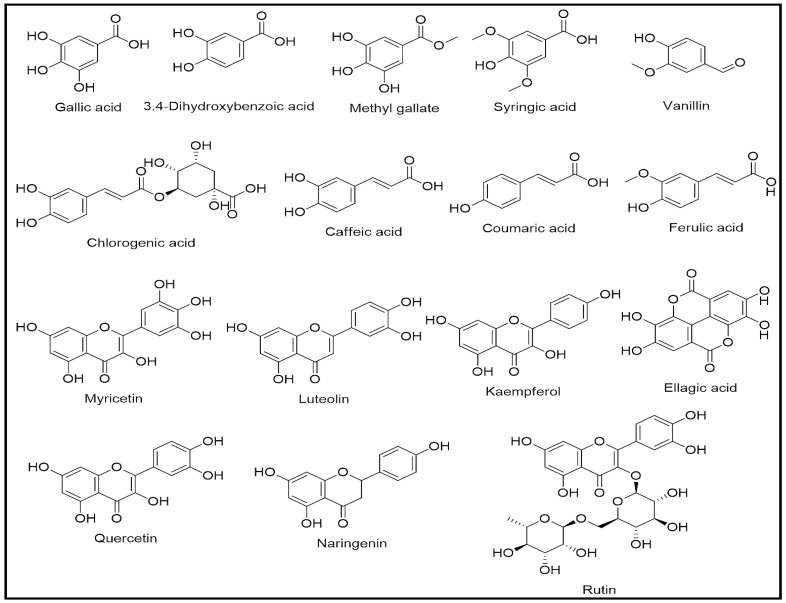
Structures of phenolics and flavonoid compounds identified from *E. chamaesyce*.

Given the strongest total phenolic and flavonoid contents as well as the best antioxidant and antimicrobial activity among all the tested plants in the current study, the phenolics and flavonoid profiling analysis was conducted for the *E. chamaesyce* ethanolic extract. The LC-ESI-MS/MS analysis identified the phenolics and flavonoids in the plant extract. Specific standards of phenolics and flavonoids were used to identify the plant constituents by matching their retention times and mass fragmentation patterns in MS (Mass) spectra with the plant’s constituents. In addition, quantitative measurements of all the identified compounds were assessed by comparing their peak areas to the known concentration standard’s peak areas in the chromatogram. Nonetheless, sixteen phenolics and flavonoid-natured compounds were identified with their mass spectral fragmentation-based anticipated structures (Figure 1, Table S2 from Supplementary Materials). Out of these, six compounds were identified as flavonoids, i.e., rutin, myricetin, luteolin, quercetin, naringenin, and kaempferol, which were totally measured at 1090.54 µg/g of the extract. Among these flavonoids, naringenin was measured at the highest concentration (505.30 µg/g), whereas myricetin was measured at the lowest concentration (2.85 µg/g). In addition, four compounds of these flavonoid classes were found of the flavonol types, i.e., rutin, myricetin, quercetin, and kaempferol; the other two were deduced to be a flavone (luteolin) and dihyroflavone (naringenin) in their structure types. Furthermore, ten phenolic constituents were identified, which represented 10427.56 µg/g of the plant extract. Among them, gallic and ellagic acids were the most abundant phenolic acids in the plant at 6716.02 µg/g and 2505.09 µg/g of the plant extracts, respectively.

### 2.4. In Silico Molecular Docking

#### 2.4.1. Binding Energies

The current investigation also looked into in silico molecular docking studies for the major constituents of the plant, as well as the most potent compound, as identified from the *E. chamaesyce* extract. The catalytic domain of TyrRS from *S. aureus* was examined to determine the binding mode of the compounds. The compounds’ affinities and their interactions with key residues were studied. The results are presented in Table S3 from Supplementary Materials. Among the 16 compounds, the best binding energy was found in ellagic acid (−10.1 kcal/mol), and the lowest binding energy was predicted for vanillin (−6.2 kcal/mol). These results demonstrated that different constituents fit well in the enzyme’s catalytic site, and experimental bioactivity exhibition may be a combined synergistic action of these compounds. It was also observed that the major compound, ellagic acid, with a 22% concentration in the plant extract, had the best binding energy among all the studied compounds. This finding supported the notion of the presence of a potential antimicrobial agent(s) in the plant extract.

#### 2.4.2. Analysis of Receptor–Ligands Interactions

**Figure 2:**
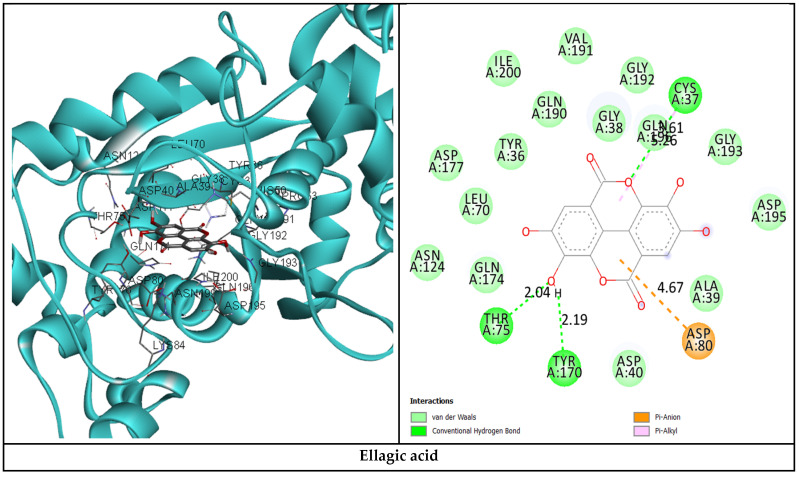

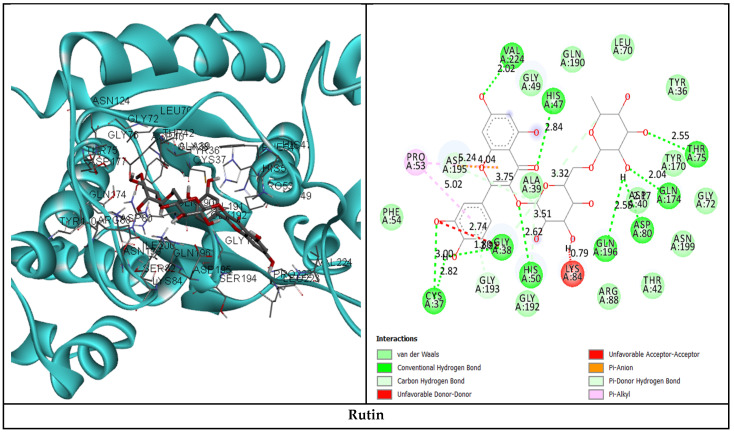
Two-dimensional (2D) and three-dimensional (3D) interactions of selected *E. chamaesyce* extract constituents with the residues of the active site of tyrosyl tRNA synthetase (PDB: 1JIJ).

The interactions between the selected ligands and the active site of TyrRS from the *S. aureus* model built from the TyrRS complex structure (PDB code: 1jij) were studied and are presented in Figure 2 and Figure S1 from Supplementary Materials. The ligands were chosen from the major constituents (gallic acid, ellagic acid) and the most potent compounds, namely, ellagic acid, rutin, luteolin, and quercetin. Ellagic acid and rutin are presented in Figure 2. Luteolin, quercetin, and gallic acid are presented in Figure S1 from Supplementary Materials. The docking study aimed to understand these compounds’ possible antibacterial activities.

Ellagic acid interactions: Ellagic acid exhibited the highest binding energy (−10.1 kcal/mol). It presented three hydrogen bonds with Thr^75^, Tyr^170^, and Cys^37^. There was one pi-anion interaction with Asp^80^. It fits very well in the active site with many Van der Waals (vdW) interactions. As mentioned before, this molecule is the second major constituent (22%), and it seemed to contribute to the potent antimicrobial activity of the plant extract.

Rutin interactions: Rutin exhibited the second-highest binding energy (−9.8 kcal/mol). It formed 10 hydrogen bonds with Cys^37^ (2 hydrogen bonds), Gly^38^, His^50^, Gln^196^, Asp^80^, Gln^174^, Thr^75^, His^47^, and Val^224^. There were two pi-(Π)-alkyl interactions with Pro^53^. There was one pi-alkyl and one pi-hydrogen donor bond interaction with Asp^195^. The molecule was also surrounded by VdW interactions.

Luteolin interactions: Luteolin (−9.7 kcal/mol) is attached to Lys^84^ by two hydrogen bonds. It is attached to Tyr^36^ by one hydrogen bond. There was one pi-alkyl interaction with Leu^70^ and one pi-anion interaction with Asp^80^. It also built other vdW interactions with the pocket residues.

Quercetin interactions: Quercetin showed binding energy of −9.7 kcal/mol, similar to the previous compound. It had four hydrogen bonds with Thr^75^, Lys^74^, Asp^177^, and Gln^174^. It had one pi-anion interaction with Asp^40^. It had one pi-alkyl interaction with Leu^70^. The remaining interactions were of VdW types.

Gallic acid: The first major compound in the *E. chamaesyce* extract was found to be gallic acid, which has a binding energy of −7.2 kcal/mol. It established two hydrogen bonds with Thr^75^ and Asp^40^. It had one pi-alkyl interaction with Leu^70^. It also had many VdW interactions.

These different results showed that several constituents of the extract, and in particular, ellagic acid, the second major compound from *E. chamaesyce* extract, had a good affinity for the active site of the enzyme and made interactions with the key residues. This suggested that these constituents contribute to the antimicrobial activity of the plant. In addition, the results showing interactions with Thr^75^, Tyr^170^, Cys^37^, Gly^38^, Leu^70^, and Asp^40^ are in agreement with a previous report [42]. The current docking results are also well supported by the literature information showing the antimicrobial activity of ellagic acid [43].

## 3. Materials and Methods

### 3.1. Chemicals and Reagents

Unless otherwise stated, all required chemicals were procured from registered vendors, including Sigma-Aldrich, USA; Oxoid Ltd., UK; and Loba Chemie Pvt. Ltd., India.

### 3.2. Plant Collection, Identification, and Extraction Process

The four halophyte aerial parts were collected from the Qassim region of Saudi Arabia (27°21′53.6″ N 42°13′21.9″ E) in March 2019 and identified as *E. chamaesyce*, *B. arabica*, *F. mollis*, and *H. salicornicum* by the taxonomist Professor Ahmed El-Oglah, Department of Biological Sciences, Yarmouk University, Jordan. The plant specimens were stored under the record numbers QPP-126–129 at the College of Pharmacy, Qassim University, Saudi Arabia. The collected aerial parts of the plants were dried under normal room conditions, ground, and extracted separately (95%, aq. ethanol, 95/5:*v*/*v*, ethanol:water). The ethanol extracts were filtered, and the solvent evaporated on a rotavapor at 40 °C.

### 3.3. TPC and TFC

TPC and TFC were measured using Folin–Ciocalteu and aluminum chloride (AlCl_3_) reagents [44]. The contents were measured as equivalents of gallic acid (GAE) and quercetin (QE). For the TPC, sodium carbonate (Na_2_CO_3_) solution (0.2 mL of a 10% concentration in distilled water (DW)) and plant extracts (1.6 mL of 0.1 mg/mL) were thoroughly mixed with 0.2 mL of the diluted Folin–Ciocalteu (1:5 in DW) reagent, and the mixture was kept at RT (room temperature) for 30 min. The absorbance of the produced blue color was measured at 760 nm wavelength. The TPC was expressed as GAE of the per gram dried extract using the GA (gallic acid) calibration curve generated from three independent experiments.

The TFC was expressed as QE of the per gram dried extract using a quercetin calibration curve from three independent experiments. In a test tube, 0.1 mL of aluminum chloride (AlCl_3_, 10% in DW), plant extracts (2 mL of 0.1 mg/mL), and 0.1 mL of potassium acetate (CH_3_CO_2_K, 0.1 mM) were added and thoroughly mixed. The reaction mixtures were stored for 30 min at RT before measuring the absorbance at 415 nm wavelength.

### 3.4. Total Antioxidant Activity (TAA) Profiles of Plant Extracts

#### 3.4.1. TAA

The TAA of the plant extracts was assayed using the known procedure [45]. Fresh molybdate standard reagent was prepared by adding 0.6 M of sulphuric acid (H_2_SO_4_) to 4 mM ammonium molybdate in a 28 mM sodium phosphate buffer. In the test tubes, 0.4 mL of the plant’s extract (200 µg) was added to 3.6 mL of the molybdate reagent. The mixture was heated to 90 °C in a water bath for 30 min. The mixture’s absorbance was recorded at 695 nm wavelength. The blank consisting of 0.4 mL of DW and 3.6 mL of molybdate reagent was used for zero performance of the spectrophotometer. The antioxidant activity of the extract was measured as equivalents of Trolox per gram of the dried plant extract.

#### 3.4.2. DPPH Scavenging Activities

DPPH, a resultant deep purple free radical scavenger, was used to assess the quenching activity of the plant extract as well as the scavenging potential of the pure compounds. The DPPH color change, which indicated the stabilization of the radicals, was used for the purpose. The calibration curve was generated using various concentrations of Trolox, a standard antioxidant reagent. Moreover, the capability of the plant extract to reduce the DPPH was estimated as equivalent to Trolox [46]. An aliquot of 1 mL, taken from 200 g of plant extract in methanol, was mixed with 1 mL of the freshly prepared DPPH free radical solution (6 mg of DPPH in 50 mL of methanol). The mixture was thoroughly mixed and set aside at RT for 30 min in the dark. The absorbance of the mixture was measured at 517 nm wavelength using methanol as the blank. The scavenging activity of the plant extract was measured as equivalent to Trolox using its standard calibration curve generated from its three independent measurements.

#### 3.4.3. FRAP Assays

The FRAP assay was used to evaluate the reducing ability of the plant extract [47]. First, 5 mL of tripyridyltriazine (TPTZ) (10 mM in 40 mM HCl), 5 mL of 20 mM ferric chloride (FeCl_3_·6H_2_O), and 50 mL of acetate buffer were mixed to prepare the FRAP reagent. Then 2 mL of the FRAP working reagent was mixed with 0.1 mL of the plant extract (200 µg of the dried extract). The mixtures were set aside for 30 min at RT before absorbances were measured at 593 nm wavelength.

#### 3.4.4. MCA Assay

The metal chelating behaviors of the plant extracts were assessed by following the Zengin et al. method, where EDTA was used as a metal-chelating standard compound to make the calibration curve [48]. The plant extract (200 µg of dried extract in ethanol) was mixed with 25 µL of ferrous chloride (2 mM) and 100 µL of ferrozine. The absorbance was measured at 562 nm wavelength against a blank (2 mL of the extract plus 200 µL of ferrous chloride without ferrozine).

### 3.5. Antimicrobial and Antibiofilm Assay

#### 3.5.1. Test Organisms

Microbes *S. aureus* ATCC 29213, MRSA, *S. saprophyticus* ATCC 43867, *S. pyogenes*-(A) ATCC 19615, *B. cereus* ATCC 10876, *E. coli* ATCC 25922, *K. pneumoniae* ATCC 27736, *P. aerugenosa* ATCC 9027, *S. typhimurium* ATCC 13311, *S. flexneri* ATCC 12022, *C. albicans* ATCC 10231, and *A. niger* ATCC 6275 were used as the test organisms for testing the antimicrobial activity of the plant extracts. All the ATCC strains were purchased from Microbiologics, Cooper Ave N, St Cloud, MN 56303, USA. At the same time, the MRSA (clinical isolate) was received from King Saud Hospital, Unaizah, Saudi Arabia. We used the same microorganisms as in the prior research; hence we did not repeat the antimicrobial susceptibility tests for these microorganisms in this study [49].

#### 3.5.2. Preliminary Antimicrobial Activity

The ethanolic extracts of the four plants, i.e., *F. mollis*, *B. arabica*, *H. salicornicum*, and *E. chamaesyce*, were used in the current study. The preliminary antimicrobial activity tests were conducted by the disc diffusion method [49,50,51,52,53]. The stock solutions of plant extracts were prepared in DMSO by diluting the plant extracts at a concentration of 250 mg/mL. The test discs were made by dispensing 20 µL of diluted plant extract (250 mg/mL) onto their respective paper discs; thus, each test paper disc contained 5 mg plant extract/disc. The negative control discs were prepared by dispensing 20 µL DMSO/disc. All test discs were sterilized by ultraviolet (UV) irradiation for 20 min before use. The remaining method was the same as reported in previous publications [49,50,51,52,53]. The ZIDs were measured on a millimeter (mm) scale. All experiments were conducted in triplicate. The results are presented as mm ± SD.

#### 3.5.3. MIC and MBC

The MIC and MBC of the plant extracts were determined using the resazurin-based micro broth dilution method and the standard spot inoculation method, respectively [49,50,54,55]. To prepare the stock solutions of the plant extracts, 100 mg/mL was dissolved in DMSO, and then 200 µL of each solution was added to the first column. The remaining columns (2–10) were filled with 100 µL of sterile tryptic soy broth (TSB). Column 11 was used as a negative control and was filled with 200 µL of standardized inoculum suspensions. Column 12 was used as a sterility control and was filled with 200 µL of sterile TSB. Following the double dilution method, the plant extracts were gradually diluted from columns 1 to 10, resulting in a final concentration range of 100–0.195 mg/mL.

The bacterial inocula were prepared in TSB following the CLSI guidelines; 100 µL of each inoculum was added to columns 1–10, resulting in a bacterial concentration of approximately 5 × 10^5^ CFU/mL, and the tested extract’s concentration ranged between 50 and 0.098 mg/mL. Then the plates were incubated at 35 ± 2 °C for 24 h, followed by the addition of 30 µL of 0.015% (*w*/*v*) resazurin solution with an additional 1–2 h of incubation.

The MIC was recorded as the lowest concentration of the plant extract preventing the resazurin solution’s color change (from blue to pink). At the same time, MBC was determined by plating the contents of the wells with concentrations from the MIC on sterile tryptic soy agar (TSA) plates, followed by incubating the plates at 35 ± 2 °C for 24 h, and recording the lowest concentration that did not produce isolated colonies of the tested organisms.

#### 3.5.4. MBIC and MBEC

MBIC is the lowest antimicrobial agent concentration that prevents biofilm formation in the tested organisms. MBIC was performed on all microorganisms that were susceptible to the plant extracts. The antibiofilm activity of the plant extract was assessed using a 96-well microtiter plate [49]. The tested organisms’ inocula were prepared in TSB at 0.5 MacFarland standard (1−2 × 10^8^ CFU/mL for bacteria). Each test well of a 96-well plate contained a 100 μL aliquot from the adjusted inocula. Then, 100 μL of different plant extract concentrations were dispensed into the test wells. As a result, the final concentrations for MBIC evaluation were MIC, 2 × MIC, and 4 × MIC. The blank control (BC) wells were filled with 200 μL of sterile TSB. The plates were incubated at 35 ± 2 °C for 24 h. Following incubation, each well’s supernatants were gently decanted by inverting the plates on a tissue paper bed. After drying in RT air for 30 min, the plates were stained with 0.1% (*w*/*v*) crystal violet at RT for 30 min before being rinsed three times with DW. The crystal violet was then solubilized by adding 200 µL of 95% ethanol to each test well. The absorbance was recorded in a microplate reader (xMark™ Microplate Absorbance Spectrophotometer-Bio-Rad, Hercules, CA, USA) at 650 nm. Each test was carried out in triplicate. The means of three independent tests were taken, and the results were expressed in mg/mL. The MBEC is defined as the lowest effective concentration (EC) of an antimicrobial agent that completely eradicates the biofilm formation by the test organisms [49]. Each test well of a flat-bottom 96-well microtiter plate was inoculated with 200 μL of inoculum, equivalent to 0.5 MacFarland standard (1−2 × 10^8^ CFU/mL for bacteria) for each test organism. The plates were incubated at 35 ± 2 °C for 48 h for biofilm formation for bacteria. The contents of the test wells were removed after biofilm formation by inverting the plates over a tissue paper bed to remove non-adherent cells. Various concentrations of the plant extract, such as MIC, 2 × MIC, and 4 × MIC, were applied to different test wells (200 μL/well). The inoculated plates were re-incubated for 24 h at 35 ± 2 °C. After incubation, plates were inverted on a tissue bed to remove test-well contents. After 30 min of RT air drying, 200 μL of sterile TSB was dispensed into each test well. Afterwards, each test well received 30 μL of 0.015% (*w*/*v*) resazurin dye. The plates were re-incubated for another 1–2 h. The MBEC values were obtained after re-incubation by observing the color change from blue to pink. The columns with no color changes (blue resazurin color remained constant) scored an MBEC.

### 3.6. Liquid Chromatography Mass Spectroscopy (LC-MS) Analysis

The analysis of *E. chamaesyce* extract was achieved using liquid chromatography–electrospray ionization–tandem mass spectrometry (LC-ESI-MS/MS) with an ExionLC AC system for separation and a SCIEX Triple Quad 5500 + MS/MS system equipped with electrospray ionization (ESI) for detection [56,57]. The separation was performed using a ZORBAX SB-C_18_ Column (4.6 × 100 mm, 1.8 µm). The mobile phases consisted of two eluents: (A) 0.1% formic acid in water; and (B) acetonitrile (LC grade)**.** The mobile phase was programmed as follows: 2% B from 0 to 1 min, 2–60% B from 1 to 21 min, 60% B from 21 to 25 min, and 2% B from 25.01 to 28 min. The flow rate was 0.8 mL/min, and the injection volume was 3 µL. For MRM analysis of the selected polyphenols, positive and negative ionization modes were applied in the same run with the following parameters: curtain gas: 25 psi; ion spray voltage: 4500 and −4500 for positive and negative modes, respectively; source temperature: 400 °C; ion source gas 1 and 2 were 55 psi with a declustering potential: 50; collision energy: 25; collision energy spread: 10.

### 3.7. Molecular Docking

In order to rationalize the favored orientation of the ligands in the active site of the receptor, the interactions between the natural compounds and *S. aureus tyrosyl*-tRNA synthetase were studied by molecular docking. The Protein Data Bank for *S. aureus* tyrosyl-tRNA synthetase (PDB code 1JIJ) was used for the crystal structure of the enzyme model. All H_2_O molecules and the co-crystallized ligand were removed from the models. Gasteiger charges and Polar hydrogens were added using AutoDockTools1.5.2 (ADT), and the file format PDBQT was prepared [58]. ADT was used to choose the grid for the docking. The grid box site in 1JIJ was centered at x: −10.908, y: 14.432, and z: 86.420 Å coordinates. The coordinates x, y, and z were 25 Å for the grid box size with a spacing of 0.375 Å. The geometries of the formed natural complexes were optimized using a conjugate gradient AMMP [59]. The transformation of the PDB file to PDBQT was performed using ADT. To perform the docking simulations, we used AutoDock Vina software [58] applying an exhaustiveness parameter of 32. ADT was applied for the analysis of the docking and conformations. Ligand–receptor interactions were explored using Discovery Studio Visualizer [59].

## 4. Conclusions

The four major native halophytes from the Saudi Arabian central regions showed different levels of antioxidant and antimicrobial activities, which seemingly were caused because of the varying levels of the secondary metabolites-based phytochemical constituents present in these plants. The plants’ ethanolic extracts were investigated for the presence of naturally abundant polyphenols and flavonoid contents in these plants. Various content analysis assays and LC-MS-based specific analyses of the most potent plant, *E. chamaesyce*, confirmed the potential of this halophyte compared to other plants used in this study. Interestingly, the plant *E. chamaesyce*, rich in polyphenols and flavonoids, showed the strongest antioxidant and antimicrobial activities. The findings showed that the ethanolic extract of the plant has strong antimicrobial, antibiofilm, antioxidant, and metal-chelating properties and conclusively established and validated the traditional use of these halophytes, especially the *E. chamaesyce*, by the locals, herbal healers, and nomadic Bedouins. The bioactivities of other tested plants, as well as the differences in the presence and quantity of phytochemical constituents, along with the particular and significant uses of the plants for specific ailments by the local population, have validated the credibility, reliability, and almost the precise nature of the traditional knowledge and folklore value of these plants. However, the present study did not elaborate on the safety of the plant extracts and their further use as therapeutic agent(s). The study also did not investigate the dose-response and activity levels as well as the practice of non-standardized dose-based therapeutic applications by herbalists and the population, which needs careful scrutiny and is a subject of further investigation to ascertain the dose and safety. It is also important to consider any vulnerable side effects and interactions of the extracts with foods and other medications for harm before it can be pharmacologically safely vouched as a therapeutic agent. However, up to only a certain extent, the in silico studies on various chemical constituents of these halophytic plants, in this case for *E. chamaesyce*, have supported the bioactivity observations, although, again, no toxicity issues were addressed. Therefore, it is pertinent to investigate further the individual chemical constituent’s activity, dose regimen, and toxicity profile. Moreover, to avoid the role of synergistic effects towards the discovery and development of new drug candidates, it is desired to validate the in silico experimentally predicted antimicrobial activity proneness profile of the individual compounds. To ensure the safe and successful use of traditional plant-derived products by the local community, numerous elements must be considered, including plant sustainability, extract production and standardization, dose, and bioactivity response characteristics.

## Figures and Tables

**Table 1:** Quantitative measurements of total phenolics, flavonoids, and antioxidant activity of the plant extracts.

Plants	TPC	TFC	TAA	DPPH-SA	FRAP	MCA
*E. chamaesyce*	68.00 ± 0.07	39.23 ± 0.03	203.12 ± 0.07	74.15 ± 0.05	270.90 ± 0.56	16.28 ± 0.01
*B. arabica*	45.32 ± 0.01	17.90 ± 0.04	86.26 ± 0.09	18.46 ± 0.03	98.31 ± 0.02	16.58 ± 0.01
*F. mollis*	47.30 ± 0.03	24.79 ± 0.03	83.31 ± 0.04	23.34 ± 0.14	112.22 ± 0.01	16.34 ± 0.08
*H. salicornicum*	49.19 ± 0.03	19.33 ± 0.05	82.44 ± 0.04	25.17 ± 0.06	96.87 ± 0.02	15.80 ± 0.07

All the experiments were conducted in triplicate. The results are expressed in mean ± standard deviation (SD). TPC was calculated in mg/g GAE; TFC was calculated in mg/g QE; TAA was measured in mg TE per gram of extract; DPPH-SA (2,2-diphenyl-1-picrylhydrazyl-scavenging activity) was measured in mg TE per gram of dry extract; MCA was measured in mg EDTA equivalents per gram of the extract.

**Table 2:** Preliminary antimicrobial activity of *F. mollis*, *B. arabica*, *H. salicornicum*, and *E. chamaesyce* plant extracts.

Microorganisms	Plant Extracts vs. ZID (mm ± SD)				
	*E. chamaesyce*	*B. arabica*	*F. mollis*	*H. salicornicum*	*DMSO*
Gram-positive bacteria					
*S. aureus* ATCC 29213	8.5 ± 0.3	8.0 ± 0.2	0.0 ± 0.0	7.5 ± 0.2	0.0 ± 0.0
MRSA	8.1 ± 0.2	0.0 ± 0.0	0.0 ± 0.0	6.9 ± 0.2	0.0 ± 0.0
*S. saprophyticus* ATCC 43867	13.7 ± 0.3	0.0 ± 0.0	0.0 ± 0.0	12.2 ± 0.3	0.0 ± 0.0
*S. pyogenes*-(A) ATCC 19615	0.0 ± 0.0	0.0 ± 0.0	0.0 ± 0.0	0.0 ± 0.0	0.0 ± 0.0
*B. cereus* ATCC 10876	16.3 ± 0.3	9.9 ± 0.2	0.0 ± 0.0	13.4 ± 0.2	0.0 ± 0.0
Gram-negative bacteria					
*E. coli* ATCC 25922	0.0 ± 0.0	0.0 ± 0.0	0.0 ± 0.0	0.0 ± 0.0	0.0 ± 0.0
*K. pneumoniae* ATCC 27736	0.0 ± 0.0	0.0 ± 0.0	0.0 ± 0.0	0.0 ± 0.0	0.0 ± 0.0
*P. aerugenosa* ATCC 9027	0.0 ± 0.0	0.0 ± 0.0	0.0 ± 0.0	8.2 ± 0.3	0.0 ± 0.0
*S. typhimurium* ATCC 13311	0.0 ± 0.0	0.0 ± 0.0	0.0 ± 0.0	0.0 ± 0.0	0.0 ± 0.0
*S. flexneri* ATCC 12022	18.1 ± 0.2	0.0 ± 0.0	0.0 ± 0.0	13.3 ± 0.3	0.0 ± 0.0
Fungal strains					
*C. albicans* ATCC 10231	0.0 ± 0.0	0.0 ± 0.0	0.0 ± 0.0	0.0 ± 0.0	0.0 ± 0.0
*A. niger* ATCC 6275	0.0 ± 0.0	0.0 ± 0.0	0.0 ± 0.0	0.0 ± 0.0	0.0 ± 0.0

Note: *S. saprophyticus* = *Staphylococcus saprophyticus*; *S. pyogenes* = *Streptococcus pyogenes*; *E. coli* = *Escherichia coli*; *K. pneumoniae* = *Klebsiella pneumoniae*; *P. aerugenosa* = *Pseudomonas aerugenosa*; *S. typhimurium* = *Salmonella typhimurium*; *C. albicans* = *Candida albicans*; *A. niger* = *Aspergillus niger*. Each test was performed in triplicate. All the results are expressed in mean ± SD. The 0.0 ± 0.0 mm indicates no antimicrobial activity.

**Table 3:** MIC, MBC, MBIC, and MBEC results of *E. chamaesyce* extract.

Microorganisms	*E. chamaesyce* (mg/mL)			
	MIC	MBC	MBIC	MBEC
*S. aureus* ATCC 29213	12.50	25.00	25.00	50.00
MRSA	12.50	25.00	25.00	50.00
*S. saprophyticus* ATCC 43867	25.00	50.00	50.00	100.00
*B. cereus* ATCC 10876	12.50	25.00	25.00	50.00
*S. flexneri* ATCC 12022	12.50	25.00	25.00	50.00

**Table 4:** MIC, MBC, MBIC, and MBEC results of *B. arabica* extract.

Microorganisms	*B. arabica* (mg/mL)			
	MIC	MBC	MBIC	MBEC
*S. aureus* ATCC 29213	25.0	50.0	50.0	100.0
*B. cereus* ATCC 10876	12.5	25.0	25.0	50.0

**Table 5:** MIC, MBC, MBIC, and MBEC results of *H. salicornicum* extract.

Microorganisms	*H. salicornicum* (mg/mL)			
	MIC	MBC	MBIC	MBEC
*S. aureus* ATCC 29213	1.56	3.13	3.13	6.25
MRSA	3.13	6.25	6.25	12.50
*S. saprophyticus* ATCC 43867	3.13	6.25	6.25	12.50
*B. cereus* ATCC 10876	0.78	1.56	1.56	3.13
*P. aerugenosa* ATCC 9027	12.50	25.00	25.00	50.00
*S. flexneri* ATCC 12022	12.50	25.00	25.00	50.00

## Data Availability

This manuscript contains all the data related to this study.

## Supplementary Materials

The following supporting information can be downloaded at: https://www.mdpi.com/article/10.3390/antibiotics12030501/s1. Figure S1: Two-dimensional (2D) and three-dimensional (3D) interactions of selected *E. chamaesyce* extract constituents with the residues of the active site of tyrosyl tRNA synthetase (PDB: 1JIJ); Table S1: One-way ANOVA for the preliminary antimicrobial potential of *E. chamaesyce*, *H. salicornicum*, and *B. arabica* extracts. Table S2: LC-MS analysis of the peel and pulp extracts of *E. chamaesyce*; Table S3: Binding energies of *E. chamaesyce* extract constituents with TyrRS receptor (PDB: 1JIJ).
