# Supplementary material for: Antimicrobial, Antibiofilm, and Antioxidant Potentials of Four Halophytic Plants, Euphorbia chamaesyce, Bassia arabica, Fagonia mollis, and Haloxylon salicornicum, Growing in Qassim Region of Saudi Arabia: Phytochemical Profile and In Vitro and In Silico Bioactivity Investigations
Source: Antibiotics (Basel). 2023 Mar 2;12(3):501. doi: 10.3390/antibiotics12030501 (PMC10044527; doi:10.3390/antibiotics12030501)
Supplement: Supplementary file 1 [file antibiotics-12-00501-s001.zip › antibiotics-2250024-supplementary.pdf]

Supplementary File

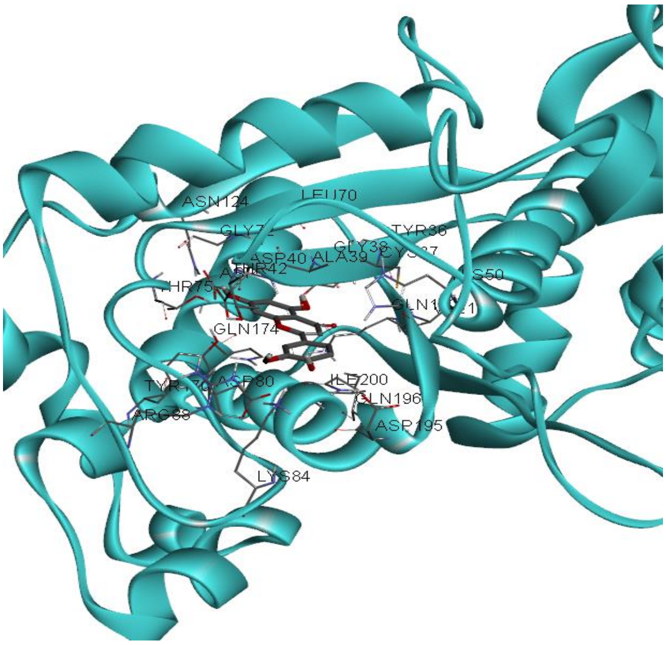

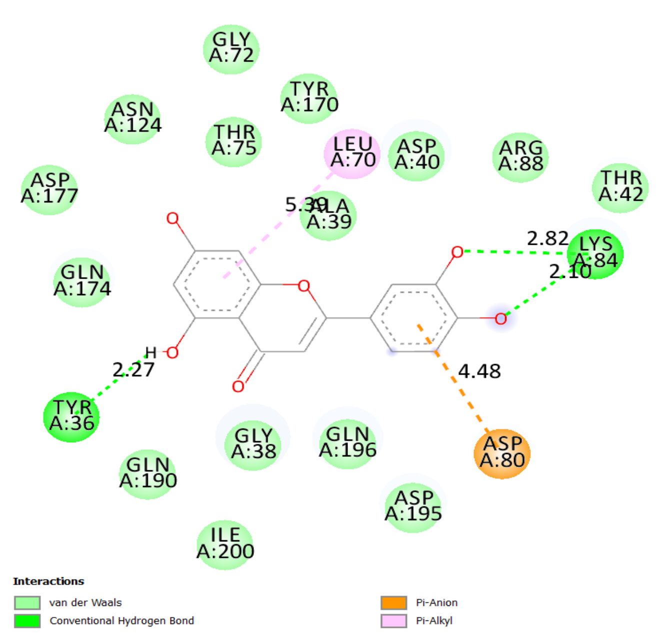

<u>Luteolin</u>

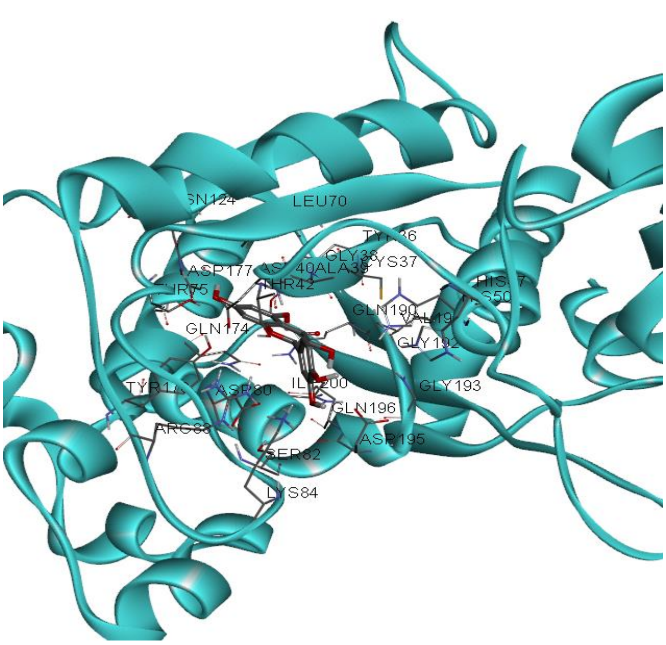

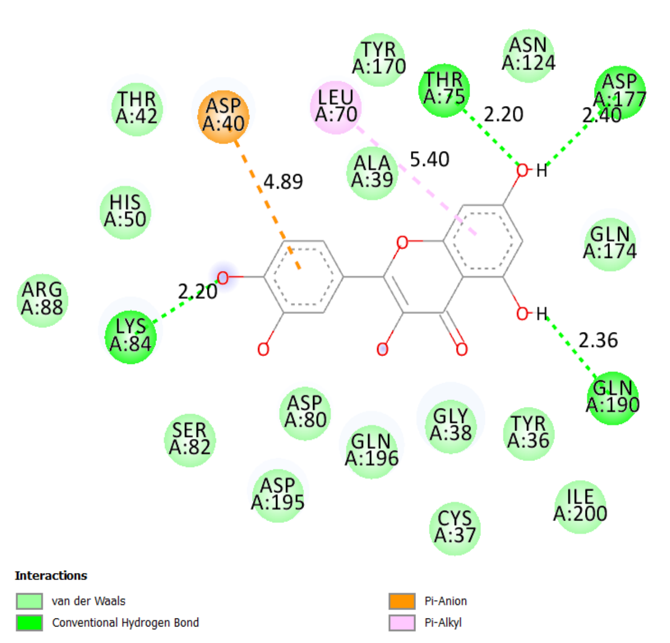

<u>Quercetin</u>

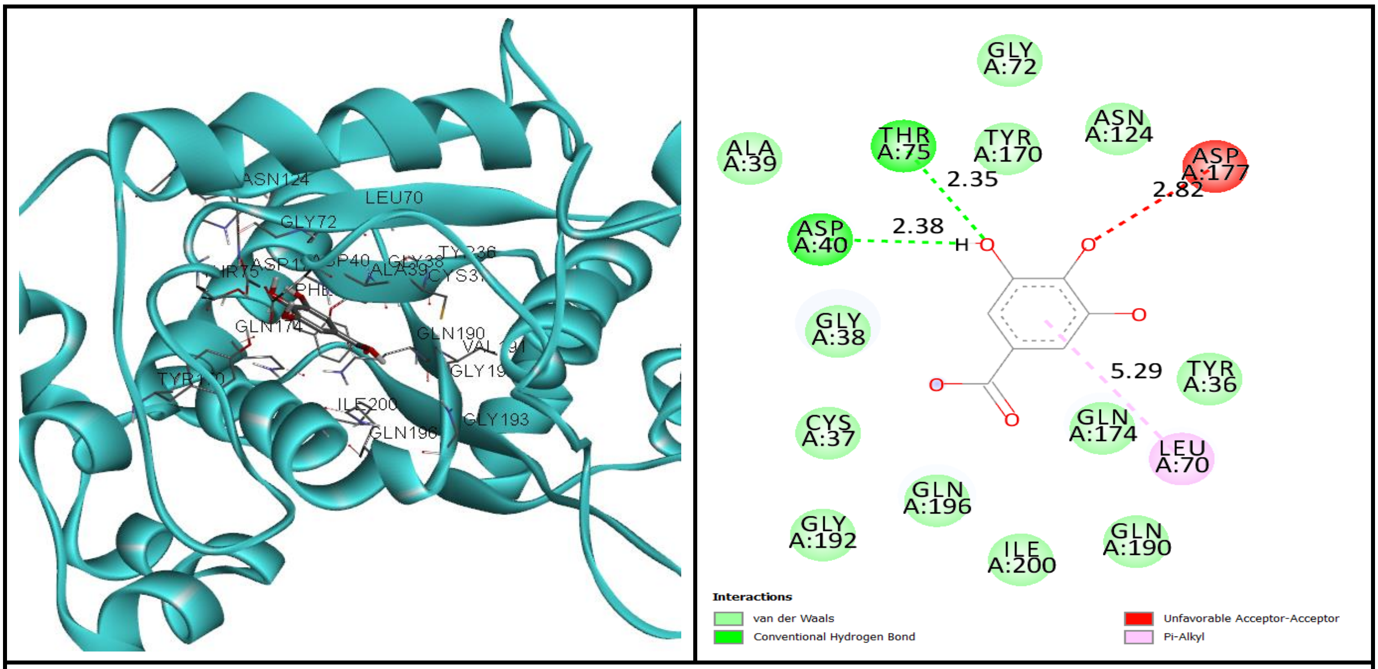

<u>Gallic acid</u>

Figure S1. Two-dimensional (2D) and three-dimensional (3D) interactions of selected *E. chamaesyce* extract constituents, luteolin, quercetin and gallic acid with the residues of the active site of tyrosyl tRNA synthetase (PDB: 1JIJ).

Table S1. One-way ANOVA for the preliminary antimicrobial potential of *E. chamaesyce, H. salicornicum,* and *B. arabica* extracts.

ANOVA						
		**Sum of Squares**	**df**	**Mean Square**	**F**	**Sig.**
E. chamaesyce	Between Groups	666.327	11	60.575	2506.561	.000
	Within Groups	.580	24	.024		
	Total	666.907	35			
H. salicornicum	Between Groups	300.366	11	27.306	756.165	.000
	Within Groups	.867	24	.036		
	Total	301.232	35			
B. arabica	Between Groups	48.927	11	4.448	667.193	.000
	Within Groups	.160	24	.007		
	Total	49.087	35			

Table S2. LC-MS analysis of the peel and pulp extracts of *E. chamaesyce*.

**Compounds**	**Retention time**	**m/z [M-H]$^{-}$**	**MS/MS**	**Concentration μg/g**	**Relative percentages %**
Gallic acid (GA)	3.84	168.9	74, 125	6716.02	58.31
3.4-Dihydroxybenzoic acid	5.72	152.9	108, 109	111.76	0.97
Chlorogenic acid	7.34	355.1	135, 163	19.73	0.17
Methyl gallate	7.45	183	78, 106, 124	981.37	8.52
Caffeic acid	8.05	178	135	17.07	0.15
Syringic acid	8.41	196.8	95, 123, 167, 182, 197	10.83	0.09
Coumaric acid	9.52	162.9	119, 135	6.95	0.06
Vanillin	9.56	151	107, 136, 151	8.00	0.07
Rutin	9.72	609	151, 179, 271, 301	241.52	2.10
Ellagic acid	9.9	301	129, 145, 157, 229, 284	2505.09	21.75
Ferulic acid	10.25	192.8	117, 133.9, 149, 178	50.74	0.44
Myricetin	11.71	317	109, 137, 151, 179	2.85	0.02
Luteolin	13.51	284.7	107, 133, 149, 175, 217	10.93	0.09
Quercetin	13.58	301	65, 121, 151, 179	154.27	1.34
Naringenin	14.99	271	65, 107, 119, 151	505.3	4.39
Kaempferol	15.36	284.7	93, 159, 227, 257	175.67	1.53
Total concentration				11518.1	

Table S3. Binding energies of *E. chamaesyce* extract constituents with TyrRS receptor (PDB: 1JIJ).

**Compound**	**Free binding energy (kcal/mol)**
Gallic acid (GA)	-7.2
3.4-Dihydroxybenzoic acid	-6.9
Chlorogenic acid	-9.1
Methyl gallate	-7.4
Caffeic acid	-7.1
Syringic acid	-6.9
Coumaric acid	-6.4
Vanillin	-6.2
Rutin	-9.8
Ellagic acid	-10.1
Ferulic acid	-6.8
Myricetin	-9.5
Luteolin	-9.7
Quercetin	-9.7
Naringenin	-9.3
Kaempferol	-9.4
